# An Exploration into Human–Computer Interaction: Hand Gesture Recognition Management in a Challenging Environment

**DOI:** 10.1007/s42979-023-01751-y

**Published:** 2023-06-12

**Authors:** Victor Chang, Rahman Olamide Eniola, Lewis Golightly, Qianwen Ariel Xu

**Affiliations:** 1 Aston University, Aston St, Birmingham, B4 7ET UK; 2 Teesside University, Campus Heart, Southfield Rd, Middlesbrough, TS1 3BX UK

**Keywords:** Hand recognition, Human–computer interaction, Machine learning, Convolutional neural network (CNN)

## Abstract

Scientists are developing hand gesture recognition systems to improve authentic, efficient, and effortless human–computer interactions without additional gadgets, particularly for the speech-impaired community, which relies on hand gestures as their only mode of communication. Unfortunately, the speech-impaired community has been underrepresented in the majority of human–computer interaction research, such as natural language processing and other automation fields, which makes it more difficult for them to interact with systems and people through these advanced systems. This system’s algorithm is in two phases. The first step is the Region of Interest Segmentation, based on the color space segmentation technique, with a pre-set color range that will remove pixels (hand) of the region of interest from the background (pixels not in the desired area of interest). The system’s second phase is inputting the segmented images into a Convolutional Neural Network (CNN) model for image categorization. For image training, we utilized the Python Keras package. The system proved the need for image segmentation in hand gesture recognition. The performance of the optimal model is 58 percent which is about 10 percent higher than the accuracy obtained without image segmentation.

## Introduction

British Sign Language recognition is a project based on the notion of image processing and machine learning classification. There has been much study in the last year on gesture recognition utilizing various machine learning and deep learning approaches without fully explaining the methods used to get the results. This study focuses on lowering the cost and increasing the resilience of the suggested system using a mobile phone camera while also detailing the steps used to conclude.

Management is a collection of operations (including planning and decision-making, organizing, directing, and supervising) aimed toward an organization’s resources (human, financial, physical, and informational) to attain organizational goals effectively and efficiently [10]. Unquestionably, good management is one that the business can depend on in the face of new and unexpected difficulties. Nevertheless, socioeconomic, political, and, most recently, health challenges have significantly impacted the efficacy and efficiency of management processes in modern organizations. Consequently, the internal and external elements affecting the organizational management process should be attentive to and evaluated.

Internal factors such as workplace culture, personnel, finances, and current technologies are under the influence of the company, while extrinsic variables such as politics, competitors, the economic system, clients, and the climate are beyond the management’s control but can have a significant influence on the productivity and accomplishment of the organization. Therefore, the management framework of a company must be critically assessed. As a firm with a rich history spanning more than a century (116 years), BMW was founded in 1916 in Munich, Germany. This establishment, which is a few years younger than Ford in 1903 and Rolls Royce in 1907, has developed one of the finest automobiles [45]. In this research, we critically reviewed BMW's management strategy throughout the 2008–2011 global economic crisis to determine why BMW effectively navigated the crisis while other companies flopped.

### Research Questions

This study aims to clarify and explain the following five research questions (RQs).

RQ1: What are the image processing approaches for improving picture quality and generalization of the project?

RQ2: What image segmentation techniques for separating the foreground (hand motion) from the background?

RQ3: What machine learning and deep learning approaches are available for image classification and hand gesture recognition?

RQ4: What hardware and or software is required?

RQ5: What are the benefits of the proposed approaches over currently existing methods? The result of the comparison between our method and other approaches can be used to determine what aspects of our techniques need to be improved for future study.

RQ6: What ethical issues does the initiative raise?

### Research Contributions

This study aims to make a contribution to the understanding of various approaches utilized to enhance picture quality during an imaging job. Specifically, we investigate image processing techniques such as erosion, resizing, and normalizing, as well as segmentation features like HSV color separation and thresholding. In addition, this study explores Machine Learning approaches used in picture classification projects, particularly for hand gesture recognition, and considers hitherto unutilized Machine Learning methods as potential alternatives. Then, this study evaluates the feasibility of the project based on the available materials and quantifies the model's performance in comparison to prior studies. Finally, we detect and address any ethical concerns that may arise in hand gesture recognition due to the potential impact of advanced algorithms on people. Overall, this study seeks to contribute to the field of hand gesture recognition and image processing, with the goal of improving human–computer interaction and addressing potential ethical issues.

## Related Literature

Hand Gesture Recognitions (HGRs) is a complicated process that includes many components like image processing, segmentation, pattern matching, machine learning, and even deep learning. The approach for hand gesture recognition may be divided into many phases: data collection, image processing, hand segmentation, extraction of features, and gesture classification. Furthermore, while static hand motion recognition tasks use single frames of imagery as inputs, dynamic sign languages utilize video, which provides continuous frames of varying imagery [5]. The technique for data collection distinguishes computer vision-based approaches from sensors and wearable-based systems. This section discusses the methods and strategies used by static computer vision-based gesture recognition researchers.

### Human–Computer Interaction and Hand Gesture Recognition

The recent technological breakthrough in computational capabilities has resulted in the development of powerful computing devices that affects people’s everyday lives. Humans can now engage with a wide range of apps and platforms created to solve most day-to-day challenges. With the advancement of information technology in our civilization, we may anticipate greater computer systems integrated into our society. These settings will enact new requirements for human–computer interaction, including easy and robust platforms. When these technologies are used naturally, interaction with them becomes easier (i.e., similar to how people communicate with one another through speech or gestures). Another change is the recent evolution of computer user interfaces that have influenced modern developments in devices and methodologies of human–computer interaction. The keyboard, the perfect option for text-based user interfaces, is one of the most frequent human–computer interaction devices (R. [15].

Human–Computer Interaction/Interfacing (HCI), also known as Man–Machine Interaction or Interfacing, has emerged gradually with the emergence of advanced computers [11, 12, 19]. HCI is a field of research involving the creation, analysis, and deployment of interacting computer systems for human use and the investigation of the phenomena associated with the subject [11]. Indeed, the logic is self-evident: even the most advanced systems are useless until they have been operated effectively by humans. This foundational argument summarizes the two crucial elements to consider when building HCI: functionality and usability [19]. A system’s functionality is described as the collection of activities or services it delivers to its clients. Nevertheless, the significance of functionality is apparent only if it becomes feasible for people to employ it effectively. On the other hand, the usability of a system with a feature refers to the extent and depth to which the system can be utilized effectively and adequately to achieve specific objectives for the user. The real value of a computer is attained when the system’s functionality and usability are adequately balanced [11, 12].

**Fig. 1:**
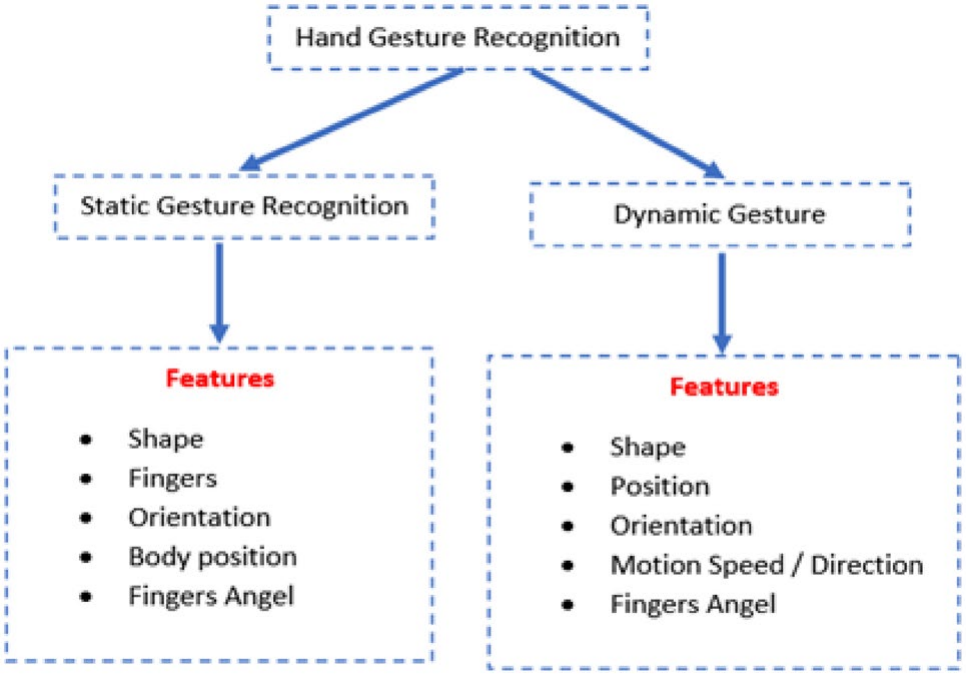
Features of hand gesture recognition

Hand Gesture Recognition (HGR) is a critical component of Human–Computer Interaction (HCI), which studies computer technology designed to understand human commands. Interacting with these technologies is made simpler when they are conducted in a natural manner (i.e., just as humans interact with each other using voice or gestures). Nonetheless, owing to the influence of illumination and complicated. Backgrounds, most visual hand gesture detection systems mainly function in a limited setting. Hand gestures are a kind of body language communicated via the center of the palm, finger position, and hand shape. Hand gestures are divided into two types: dynamic and static, as shown in Fig. 1 below. The stationary gesture relates to the fixed form of the hand, while on the other hand, the dynamic hand gesture consists of a sequence of hand motions like waving. There exist different hand motions in a gesture. For instance, a handshake differs from one individual to another and depends entirely on time and location. The main distinction between posture and gesture is that the former focuses on the form of the hand, while the latter focuses on the hand motion.

The fundamental objective of gesture recognition research is to develop a technology capable of recognizing distinct human gestures and utilizing them to communicate information or control devices [28]. As a result, it incorporates monitoring hand movement and translation of such motion as crucial instruction. Furthermore, Hand Gesture Recognition methods for HCI systems can also be classified into two types: wearable-based and computer vision-based recognition [30]. The wearable-based recognition approach collects hand gesture data using several sensor types. These devices are mounted to the hand and record the position and movement of the hand. Afterwards, the data are analyzed for gesture recognition [30, 38]. Wearable devices allow gesture recognition in different ways, including data gloves, EMG sensors, and Wii controllers. Wearable-based hand gesture identification systems have a variety of drawbacks and ethical challenges: covered later in this paper.

**Fig. 2:**
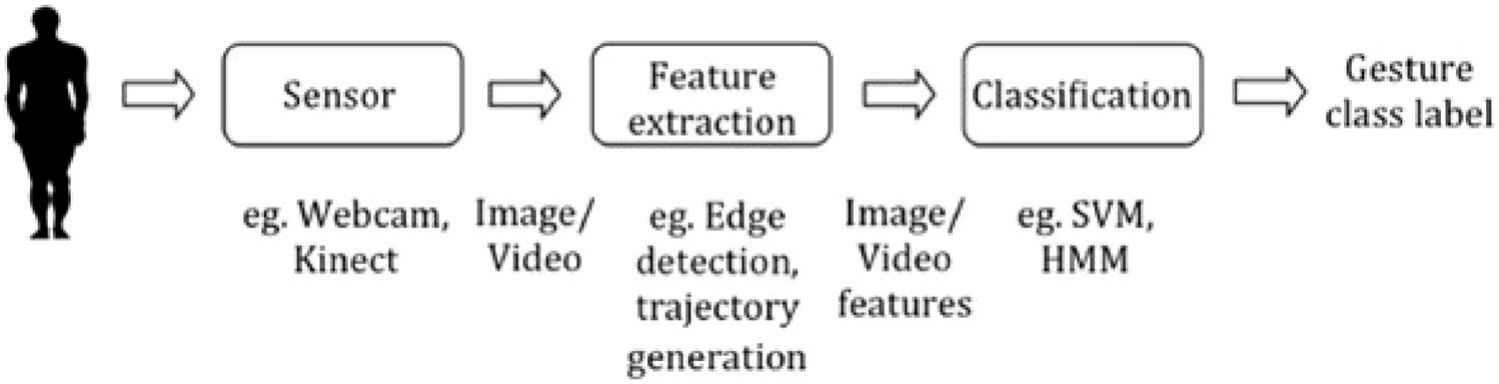
Computer vision-based gesture recognition

In contrast, computer vision-based solutions are a widespread, appropriate, and adaptable approach that employs a camera to capture imagery for hand gesture recognition and enable contactless communication between people and computers [30, 38]. Moreover, the vision-based recognition technique uses different image processing techniques to obtain the hand position and movement data. This method detects gestures based on the shapes, positions, features, color, and hand movements (Fig. 2). However, vision-based recognition has certain limitations in that it is impacted by depending on the light and crowded surroundings [38].

### Image Processing

The human eye can perceive and grasp the things in a photograph. Accurate algorithms, and considerable training, are necessary to make computers comprehend like people [13, 14, 16]. Image data account for about 75 percent of the information acquired by an individual. When we receive and use visual information, we refer to this as vision, cognizance, or recognition. However, when a computer collects and processes visual data, this is called image processing and recognition. The median and Gaussian filters are two prevalently used filtering techniques for minimizing distortion in collected images [5]. Zhang et al. [46] adopted the median filter approach to remove noise from the gesture image to generate a more suitable image for subsequent processing. Also, Piao et al. [33] also presented the Gaussian and bilateral filter strategies to de-noise the image and created a more enhanced image. On the other hand, Treece [41] proposed a unique filter that claimed to have better edge and details retaining capabilities than the median filter, noise-reducing performance comparable to the Gaussian filter, and is suitable for a wide range of signal and noise kinds. Scholars have also researched other filtering algorithms. For example, Khare and Nagwanshi [21] presented a review of nonlinear filter methods that may be utilized: for image enhancement. They conducted a thorough investigation and performance comparison of the Histogram Adaptive Fuzzy (HAF) filter and other filters based on PSNR (Peak Signal to Noise Ration).

Morphological transformation is another image processing procedure often used to eliminate undesirable content from an image. A notable example is H. Hassanpour et al. (2015), which use morphological transformations to improve the quality of different medical photographs. Moreover, Morphological transformation has been utilized in many studies to extract features and contour areas critical for recognition or classification tasks [6, 39, 43]. Lastly, Histogram Equalization is another image processing technique for image enhancement that has received considerable attention. Xie et al. [44] investigated the basic concept of histogram equalization for image enhancement and showed that histogram equalization could enhance image effect and contrast. Abdullah-Al-Wadud et al. [1] also addressed a dynamic histogram equalization (DHE) method that splits the histogram relying on local minima and allocates specific grey level ranges for each partition before equalizing them individually, taking control over the impact of classical HE so that it performs image enhancement without sacrificing detail. Abdullah-Al-Wadud et al. [1] asserted that the DHE technique outdoes other current methods by improving contrast without presenting adverse effects or unacceptable artifacts.

### Image Segmentation

The segmentation stage entails splitting images into numerous separate regions to separate the Region of Interest (ROI) from the rest of the imagery. Scholars have discussed different methods for image segmentation discussed below. Skin color segmentation is typically done in different color spaces, depending on the image type and content. Muhammad and Abu-Bakar [27] suggested a color space blend of HSV and YCgCr for skin detection and segmentation that responds well to various skin color tones while being less sensitive to pixels in the background that look like the skin.

Shaik et al. [37] conducted a thorough literature review, displaying various color spaces used for skin color identification, and discovered that RGB color space is not favored for color-based identification and color assessment due to the blending of color (chrominance) with the level of intensity (luminance) data and its non-uniform features. Furthermore, Shaik et al. [37] argued that Luminance and Hue-based strategies actively discriminate color and intensity level even under bad lighting conditions, a claim backed by their experimental results, demonstrating the YCbCr color space performance in the segmentation and detection of skin color in color images.

Saini and Chand [35] addressed the application and retrieval of skin pixels in the RGB color model in their publication, and they demonstrated the need for changing color models by monitoring the impacts of variables such as noise and illumination conditions. Furthermore, they debated various color models that are: commonly utilized in research, such as the HIS, HSV, TSL, and YUV color spaces. Saini and Chand [35] also speculated that the presence of illumination, shadows, and interference could impact the appearance of skin color and make segmentation and detection difficult. As a result, an RGB-based skin segmentation method for retrieving skin pixels was introduced in their research study, along with a computerized method for automatically transitioning color models in different color spaces, such as RGB into HSV or vice versa, to obtain the best noticeable image pixels.

Other methods, aside from skin color-based segmentation, have been extensively researched in the literature. Phung et al. [32] examined the pixel-wise skin segmentation technique based on color pixel classification, revealing that the Bayesian classifier based on the histogram technique and the multi-layered perception performed better than other methods, including the piece-wise linear and the Gaussian classifiers. Additionally, they argued that the Bayesian classifier combined with the histogram method is practical for the skin color pixels classifying issue due to the low dimension of the feature space and the availability of an enormous training set. They note, nonetheless, that the Bayesian classifier consumes far more memory than the MLP or other algorithms. Concerning color representations, their investigation using a Bayesian classifier demonstrates that the selection of color model does not affect pixel-wise skin segmentation. They concluded, nevertheless, that using chrominance channels solely reduces segmentation results and that there are considerable efficiency differences across various chrominance options.

### Gesture Recognition and Machine Learning Algorithms

Various machine learning and deep learning algorithms have recently been utilized for hand gesture recognition and classification of static and dynamic gestures. Different machine learning algorithms have been utilized for static hand gesture recognition [7, 25, 29]. Liu et al. [25] introduced Hu moments and support vector machine-based approaches (SVMs). Firstly, Hu invariant moments are retrieved: into a seven-dimensional vector. Secondly, an SVM classifier is utilized to determine a decision boundary between the integrating and flawed hands. On the other hand, Feng and Yuan [7] and Nagashree et al. [29] retrieved the histogram of gradients (HOG) for feature extraction and the Support Vector Machine (SVM) classifier, which is extensively utilized for classification to train these relevant attributes. At testing time, a decision is made using the earlier learned SVMs, and the same gesture recognition rate with a comparison in distinct illumination scenarios. The findings reveal that the HOG feature extraction and multivariate SVM classification approaches have a substantial recognition accuracy, and the system is more resistant to lighting.

An Artificial Neural Network (ANN) is a computer processing technology with functional properties like human neural systems. In ANN for Hand gesture recognition, we studied several works of literature [8, 17, 31].

Oyedotun and Khashman [31] suggested using a deep convolutional neural network to solve the challenge of hand gesture recognition for all 24 hand gestures from Thomas Moeslund’s gesture recognition repository. They demonstrated that more biologically oriented DNN, such as the convolutional neural network and the stacked de- noising autoencoder, can grasp the complicated hand gesture identification challenge with reduced misclassification. Islam et al. [17] reported a static hand gesture recognition approach based on CNN. Data augmentation techniques such as re-scaling, resizing, shearing, translation, width, and height altering were applied: to the pictures used to train the model. Flores et al. [8] suggested techniques for recognizing the static hand gesture alphabet of the Peruvian sign language (LSP). They used image processing methods to remove or minimize noise, boost contrasts under different lighting conditions, segment the hand from the image background and ultimately recognize and trim the area holding the hand gesture. They used convolutional neural networks (CNN) to categorize the 24 hand gestures, creating two CNN designs with varying numbers of layers and attributes for every layer. The testing revealed that the initial CNN before data augmentation has a lower accuracy than the one after.

The timing mismatch makes it impossible to match two different gestures in dynamic gesture recognition using Euclidean space. Nevertheless, scholars have developed sophisticated techniques and algorithms for detecting and identifying dynamic hand gestures in real-time [23, 24, 40]. [40] created a method that uses picture entropy and density clustering to exploit critical frames from hand gesture video for additional feature extraction, potentially improving identification efficiency. Furthermore, they presented a pattern fusion technique to increase feature representation and enhance the system performance.

Lai and Yanushkevich [24] suggested a hybrid of convolutional neural networks (CNN) and recurrent neural networks (RNN) for automatic hand gesture identification utilizing depth and skeletal data. In their study, Recurrent neural networks functioned effectively in recognizing sequences of motion for every skeleton joint provided the skeleton details, while CNN was used to retrieve spatial data from depth images. Köpüklü et al. [23] suggested a two-level hierarchical system of a sensor and a classifier that allows offline-working convolutional neural network (CNN) models to run online effectively using the matching template technique. They used several CNN designs and compared them in terms of offline classification accuracy, set of variables, and computing efficiency. They employed Levenshtein distance as an assessment tool to assess the identified gestures' single-time activations.

### Applications of Hand Gesture Recognition

Hand gesture recognition has several applications in various industries, such as virtual worlds, robotics, intelligent surveillance, sign language translation, and healthcare systems. The section below delves more into a few of the application areas.

#### Applications in Healthcare

Over the years, interactive systems have expanded significantly as several research efforts have proved their value and influence in different sectors, including drug production, medicine, and healthcare, after effective experiments. Versions of interactive systems in medicine, there has been a desire for such systems to be used in the healthcare and medical fields. Therefore, countries have been interested in building many interactive systems, including medical robots, and clearly by providing financing. Moreover, scholarship opportunities to promote research and innovation in the Human–Computer Interaction sector [36].

The continual progress of this kind of innovation is now recognized and welcomed in practically all industries. The concept of human–computer interaction systems will benefit the healthcare and medical industries, extensively utilizing the novel concepts. Yearly, new variations, designs, aesthetics, and maneuverability are developed, particularly human-inspired or humanoid robotics that can think and behave like people and act like humans. The continued advancement of this technology is lauded and has significantly influenced the medical and healthcare fields [36].

Undoubtfully, the prospect of computerized assistance will result in a significant boost in the quality of care. However, the performance and viability of such systems will be determined by how effective the interactive systems are in the medical and healthcare sector and how valuable they are to patients. Undoubtedly, the importance of doctors' confidence measures in understanding the effectiveness of the new technology systems to be deployed in the healthcare profession cannot be overstated [36].

It is essential to maintain the environment aseptic during medical surgery. However, during surgery, the physician must also see the patient’s clinical visual information via the computer, which must be sterile. Therefore, the existing method of the human–computer interface makes it difficult for workers to handle during operation. It raises the workload and the number of operational workers needed in the theatre. As a result, ensuring a speedy, accurate, and safe procedure becomes challenging.

The drawback mentioned above can be overcome using the hand gesture recognition approach. A notable example of a Hand Gesture Recognition application is the optimization of the surgical process utilizing the Kinect for Windows hardware and SDK created by a team at Microsoft Research Cambridge. The device allows clinicians to adjust, relocate, or enlarge Scans, Magnetic resonance imaging (MRI), and other medical data using simple hand motions (Douglas).

Wachs et al. [42] created a gesture-based system for sterilized viewing of radiological images, another notable example of Hand Gesture Recognition (HGR) research and application in a surgery theatre. The sterilized human–machine interaction is critical since it is how the physician handles clinical data while preventing it.

Contamination of the patient, the surgical theatre, and the accompanying doctors. The gesture-based technology might substitute touchscreen displays already used in many medical operating rooms, which must be enclosed to prevent contamination from accumulating or spreading and need flat surfaces which must be thoroughly cleaned after each treatment—but sometimes are not. With healthcare infection rates currently at alarmingly high levels, hand gesture recognition technology will be a viable option [9].

Another noteworthy application of the HGR technology is Sathiyanarayanan and Rajan’s [36] MYO diagnostics systems, which is applicable in interpreting Electromyography (EMG) patterns (graphs), bytes of vector data, and electrical data of our complex anatomy within our hand. To identify hand movement, the system employs complex algorithms that are interpreted: as instructions. The system will allow for collecting massive amounts of data and investigating a series of EMG lines to identify medical issues and hand motions.

#### Applications in Robotics

Van den Bergh et al. [3] created an automated hand gesture detection system using the available Kinect sensor. Using a sensor enables complicated three-dimensional motions while remaining resistant to disrupting objects or humans in the environment. The technology is embedded into an interactive robot (based on ROS), enabling automated interaction with the robot through hand gestures. The robot’s direction is determined by the translation of directing motions into objectives.

Robot technology has advanced considerably in recent years. However, there is a hurdle in developing the robot’s capacity to comprehend its surroundings, for which the sensor is crucial [4]. Using hand gesture recognition algorithms is advisable to manage the robot’s behavior efficiently and effectively, a new research hotspot in robot vision and control. A notable example is the Smartpal service robot, which utilizes Kinect technologies and allows users to operate the robot with their gestures, simulating the users’ actions [4]. We feel that as time progresses, using hand gestures to instruct the robot to do different tasks is no longer unrealistic for us.

#### Applications in Gaming and Virtual Realities (VE)

There has been a trend in the gaming industry toward hand gesture recognition systems, where gestures are used as instructions for video games rather than the traditional approach of touching keys on a keypad or using a controller. It is essential for these modern interfaces to recognize accidental movements and consistent gestures so that the user can have a more natural experience. Kang et al. [18] provided a unique approach to gesture identification that blends gesture detection and classification. The system distinguishes between purposeful and unintended motions within a particular visual sequence Kang et al. [18].

## Methodology

**Fig. 3:**
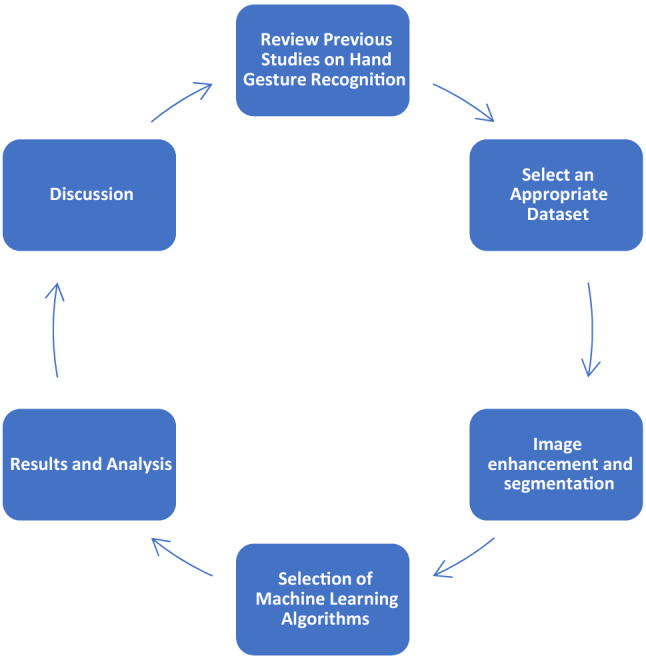
Research framework

In this study, we review previous literature in this area that has a focus on human–computer interaction and hand gesture recognition. We then select a dataset of images for analysis. In the step of image enhancement and segmentation, the quality of raw images is improved by reducing background noise, applying color space conversions, as well as isolating the main image from its background. After that, machine learning algorithms such as CNN are employed to learn the attributes and conduct hand gesture recognition. We then observe the results of the algorithms and analyze them in two parts focusing on image enhancement and hand gesture recognition. Finally, we provide a discussion of the results, including challenges and limitations, ethical considerations and opportunities for future work in this area (Fig. 3).

### British Sign Language

**Fig. 4:**
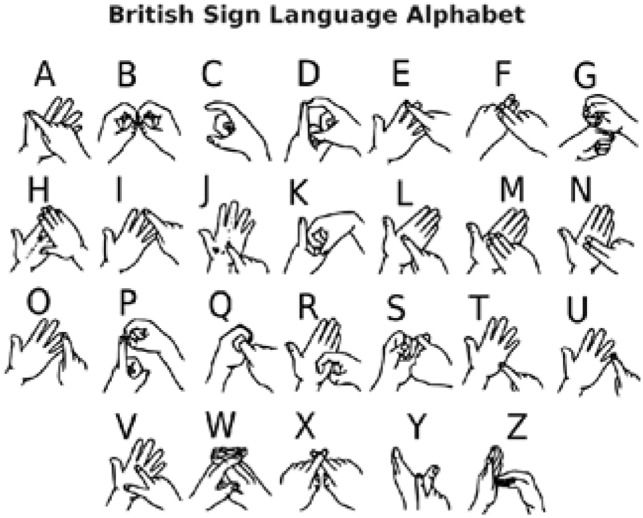
Two-hand British sign language

**Fig. 5:**
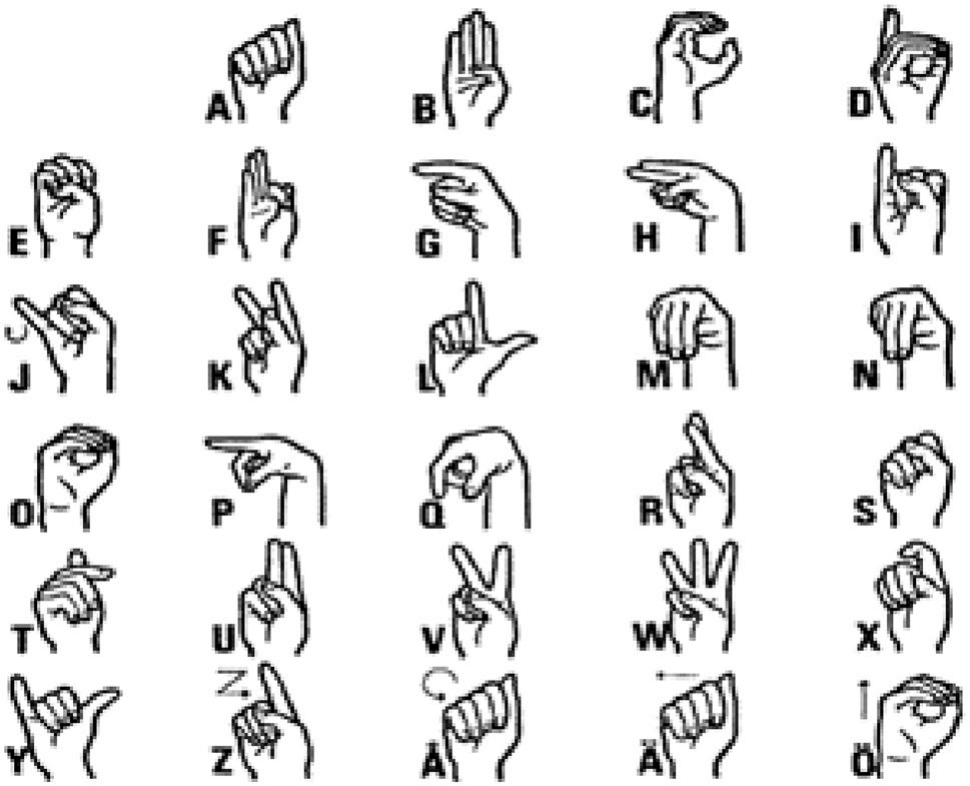
One-Hand British sign language

British Sign Language (BSL) is a visioned language used by persons with hearing or speech disabilities to convey meaning via word-level gestures, nonmanual characteristics such as facial expression and body posture, and fingerspelling (spelling words using hand movements) [26]. Figures 4 and 5 below depict the BSL alphabet with two hands and one hand, respectively. We utilized the one-hand alphabet in this project due to many features of two-hand BSL alphabets that make identification difficult.

### Dataset

In this paper, we worked on 5 one hand BSL alphabets: A, I, L, R, and V. For each of the five BSL alphabets utilized in this study, we developed a data collection of 90 distinct signs. A single signer performs each hand sign five times in varied lighting and timing circumstances. Moreover, to improve real-world performance and prevent over-fitting, we employed a validation dataset obtained under different conditions from the training dataset.

The images used in this project were captured with an iPhone 11 Pro Max camera, which has triple 12MP Ultra-Wide, Wide, and Telephoto lenses and an Ultra-Wide: 2.4 aperture and a 120° field of view. We collected two photos for the hand segmentation (color separation) task, one with and the other without a background. The images were separated and stored in separate folders. Each picture was processed and segmented before being augmented to expand the dataset for each sign. As part of the augmentations, the photos were randomly resized and rotated. The dataset was then subdivided into folders for each alphabet.

### Image Processing

The first step in every image processing system is processing raw pictures. Image processing is essential to keep all images uniform and consistent, which increases the accuracy and efficacy of the subsequent segmentation and feature extraction methods. Consequently, background noise should be decreased to enhance images and color space conversions, emphasizing the image’s region of interest better. All the images should be converted: to different color spaces and determine the optimal color space for color separation to enable image segmentation.

### Image Segmentation

Recently, many efforts have focused on the Image segmentation process, a critical stage in image processing that analyzes the digital image by segmenting it into several sections and is used to differentiate between distinct elements in an image into foreground and background based on various criteria like grey level values or to the texture [20, 34]. Image segmentation is acknowledged as the initial and most fundamental procedure in numerous computer vision tasks, including hand gesture recognition, medical imaging, robotic vision, and geographical imaging. Scholars have previously examined several segmentation approaches or algorithms in the literature. These solutions overcome several limitations of traditional hand gesture recognition systems. However, no one method can be deemed a superior approach for all types of images—such strategies are only appropriate for the particular image and purpose.

Thresholding, region growing and region merging and splitting, clustering, edge detection, and model-based approaches are the six types of image segmentation methods. All splitting approaches are based on two fundamental concepts: intensity values and discontinuity and similarity. The discontinuity progresses to image segmentation based on a sudden shift in intensity levels in the picture. In this method, we are primarily interested in recognizing distinct spots. The other strategy is centered on pixels, which are comparable in some regions according to the predefined parameters used to split images, and it comprises procedures such as Thresholding, Region expansion, and region splitting and merging.

### Color Model

A color model is a mathematical abstraction representing colors as tuples of integers with three or four values or color components. The collection of generated colors is referred to as “color space” when the color model is connected with an appropriate depiction of how the elements are to be inferred and the circumstances are observed. Color space may also be used to explain how human color vision might be simulated, which is used: in a range of applications, including computer vision, image analysis, and graphic design. Moreover, color space and color models are substantially equivalent in some cases. There are many color space bases, including the Luminance color model (YUV, YCbCr, and YIQ), Hue color model (HSI, HSV, and HSL), and the RGB color model (RGB, normalized RGB) [2, 22]. By default, the Python OpenCV library transforms pictures into BGR format. However, the BGR picture is converted into any other color space using transformative functions. RGB Color space is the most fundamental kind of picture representation, yet some applications, such as OpenCV, feel that using alternative color spaces is more convenient.

Kolkur et al. [22] stated that color space preference is the first step in skin color segmentation. Furthermore, they acknowledged that the suitable specified threshold for recognizing skin pixels in a given image might be provided by a combination of one or more color spaces. Typically, the appropriate color space is usually determined: by the skin recognition application.

### RGB Color Model

A natural image’s default color model is RGB (Red, Green, and Blue). An image is represented: by m x n × 3 arrays of color pixels in this form, where each pixel is a triplet of three colors, red, green, and blue, at a spatial position (m, n) Hema and Kannan [16] (Appendix 1). The three-color elements can be thought of as a stacking of three separate layers. Furthermore, pixels in an image have a red layer, a blue layer, and a green layer, resulting in an RGB image [16]. All these color components can be seen as a three-dimensional model. When all three-color channels have a value of 0 in additive color mixing, it signifies that no illumination is emitted, and the final color is black. The resultant color is white when all the three-color channels are set to their peak value, i.e., 255. Tv screens are excellent illustrations of how RGB color mixing is used. This color space is more susceptible to noise than others since it combines illumination and chromatic elements [2].

### HSV Color Space

HSV (Hue, Saturation, Value) color space is much closer to RGB color space, which people use to describe and interpret colors. Humans see hue as the dominating color. The quantity of white light that varies with color is referred to as Saturation. The value represents the brightness/intensity. Hue is an abbreviation for tint, Saturation is an abbreviation for shade, and Value is an abbreviation for tone. A HSV color space may be seen as a geometric cylinder, with the angular dimension representing Hue(H), beginning with primary red at 0°, progressing to primary green at 120°, primary blue at 240°, and eventually curving back to red at 360°. Saturation refers to the distance from the center axis of the HSV cylinder (S). A saturation value heading towards the outside border indicates that the colorfulness value for the color described by the hue is reaching its peak. The Value (V) is the center vertical axis of HSV color space, extending from black at the bottom with brightness or value 0 to white at the top with lightness or value 1 (Appendix 1). Figure 9 below shows that the color model in this space can be easily separated, unlike in the RGB and HSV color space discussed above. Therefore, we selected the HSV color space as the optimal color model for the segmentation based on color space.

### Color Image Segmentation Using HSV Space

**Fig. 6:**
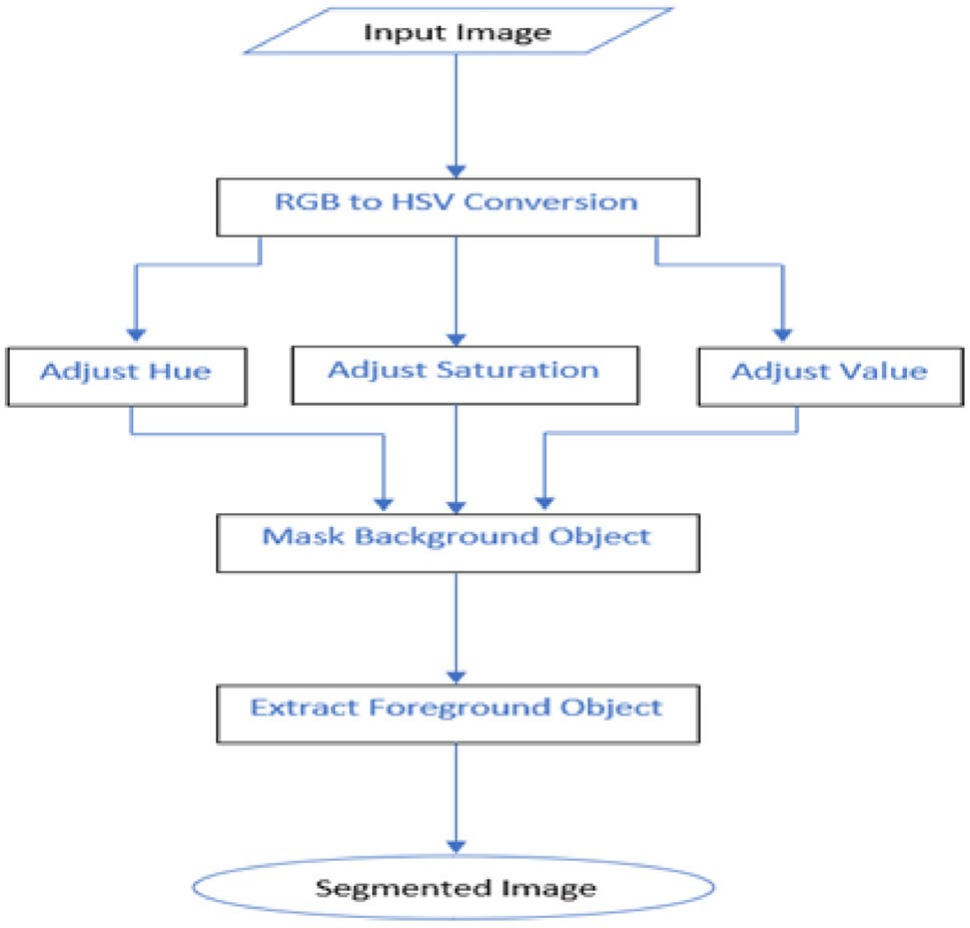
HSV-based segmentation flowchart

The detection of the color of the skin surface is an excellent example of the application of color-based image segmentation for recognizing a particular item based on its color space. A popular image segmentation phase is determining skin color via distinct color spaces. Segmenting the image’s foreground object to identify and recognize the hand area is the first step in this project's three-phase technique, shown in the flowchart in Fig. 6 below. The first stage is to choose a Region of Interest (ROI) from the provided picture; the second step is to alter the HSV values inside the ROI to extract a mask; the last step is to select the ROI using the image mask. As shown in Fig. 6, segmenting the picture in either the BGR or RGB color space is highly challenging and will not result in the optimal result, so we switched the image to HSV and investigated the segmentation potential.

How the Flowchart works is as follows. First, an RGB image is transformed into an HSV image using the HSV color space conversion algorithm. Next, the resultant HSV model components (Hue, Saturation, and Value) are separated into constituent values, as shown in Fig. 6, which are then represented in a range using the Python library. Finally, the excellent HSV value for a particular image in a specific hand gesture is determined by interactively changing the values of each Hue, Saturation, and Value component.

### Hand Gesture Recognition Algorithm

Deep Learning is quickly emerging as a prominent sub-field of machine learning owing to its exceptional performance over many data sources. Convolutional neural networks are an excellent approach to using deep learning algorithms to categorize images. The Keras Python package makes creating a CNN straightforward. CNN utilizes a multi-layer architecture that includes an input layer, an output layer, and a hidden layer composed of numerous convolutional layers, pooling layers, and fully linked layers.

#### Convolution Layer

Convolution is a mathematical operation on two functions that yields a third function that explains how the form of their form is dependent and one affects the other. Convolutional neural networks comprise several layers of artificial neurons. Artificial neurons are mathematical functions that compute the weighted sum of many inputs and output an activation value, an approximate replica of their biological counterparts. When feeding an image into a ConvNet, each layer creates many activation functions, which are passed on to the next layer. Typically, the first layer removes crucial information, such as horizontal or vertical edges. This output is sent to the subsequent layer, which recognizes more complicated characteristics like corners or combinational edges. As we get further into the system, it recognizes more sophisticated features, such as objects and characters.

#### Pooling Layer

As with the Convolutional Layer, the Pooling Layer is responsible for lowering the spatial dimension of the Convolved Feature. This reduces the CPU power necessary to analyze the data by decreasing the dimensions. Pooling is classified into two types: average Pooling and maximum Pooling. Max Pooling is a technique for determining a pixel’s highest value inside a kernel-covered picture region. Additionally, Max Pooling acts as a Noise Suppressant. It eliminates all noisy activations and conducts de-noising and dimension compression. Average Pooling produces the mean of all values in the Kernel’s section of the picture. Average Pooling is just a distortion suppression strategy that reduces dimension. As a result, we may conclude that Max Pooling outperforms Average Pooling.

## Results

The hand gesture recognition system is separated: into two tasks, which are explained lengthily below.

### Image Enhancement and Segmentation Phase

The first task is image segmentation, which includes removing the image's background contents and providing an image; without the background. Before beginning image segmentation, we must first do image processing, as described in our technique above.

#### Image Enhancement

**Fig. 7:**
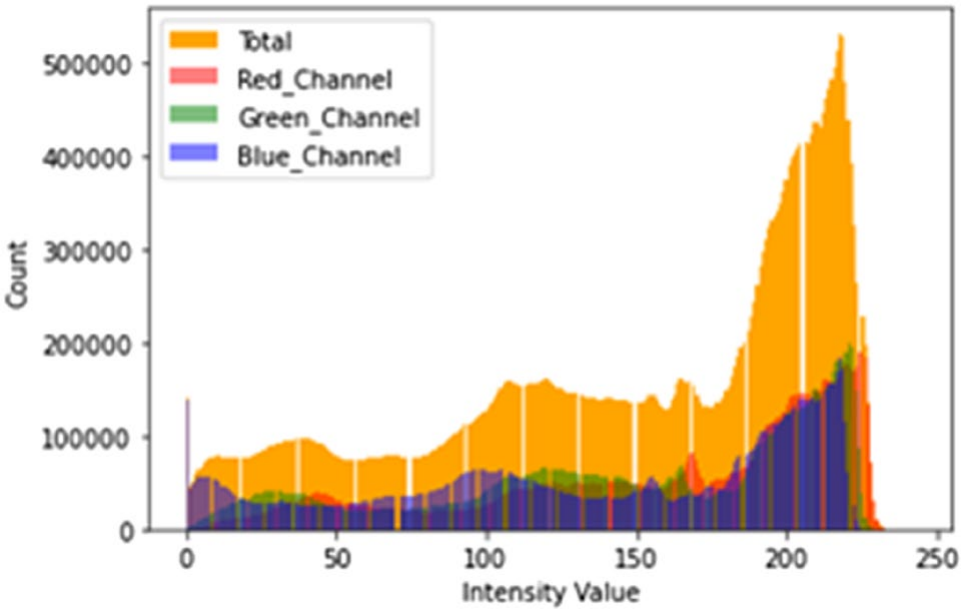
Histogram of a sample image

To start, we examine a sample of our picture for analysis using the image histogram, as shown in Fig. 7 below. The hand gesture image was loaded using the OpenCV library, which automatically converts the image into a BGR space. The difference between the RGB and BGR color spaces can be seen in Fig. 7.

**Fig. 8:**
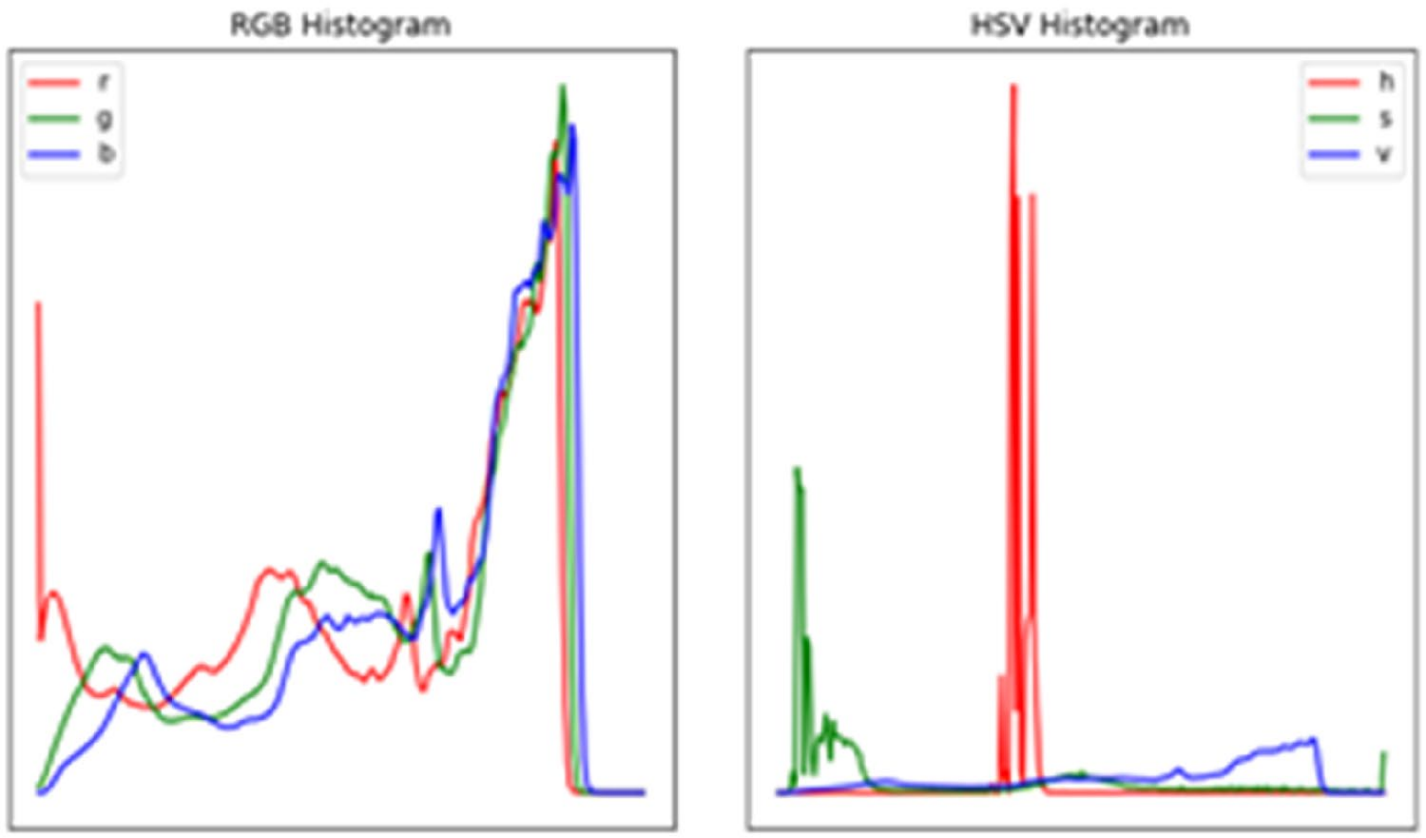
RGB and HSV histogram of a sample hand gesture

Figure 8 depicts the intensity value and count of the RGB color model from one of our dataset sample images. In addition, we produced a clearer histogram based on the RGB and HSV color spaces for better comprehension. We attempted to improve the image before moving on to the segmentation, and the task was to evaluate whether the image enhancement outcome was favorable. First, we employed several denoising algorithms to filter the picture and decrease noise. Then, we used the median and mean filter approaches on the example picture and found no difference between the original and filtered images, illustrated in Fig. 8 below.

**Fig. 9:**
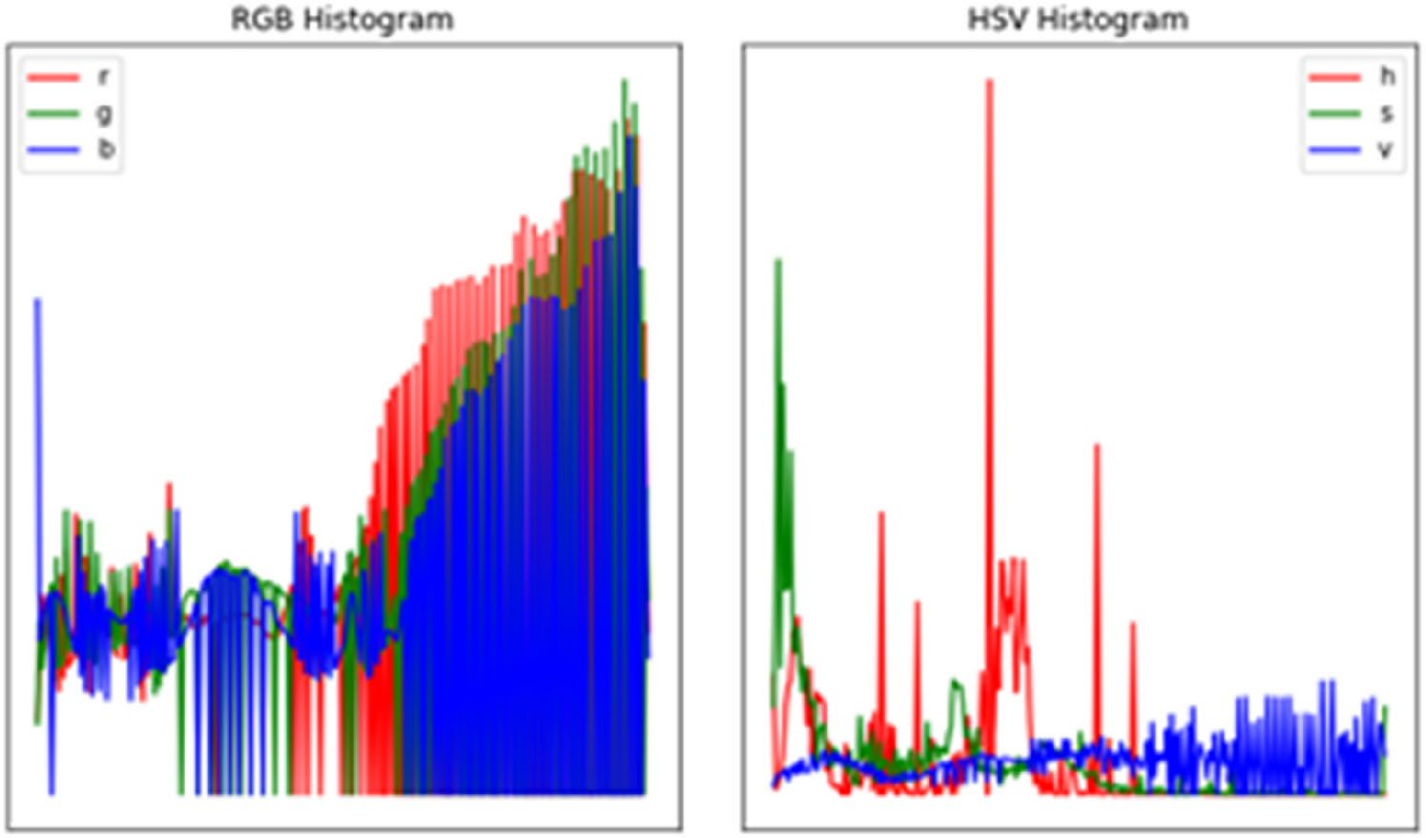
RGB and HSV histogram after equalization of a sample hand gesture (**a** left and **b** right)

Finally, we employed the Histogram of Equalization approach to improve the image quality and discovered that it did not provide the best results. Figure 9 compares the original and obtained images after Histogram Equalization. As seen in the above figure, equalization did not lead to an improved image. Therefore, we examined the histogram of the multi-channel image in the RGB and HSV color spaces to understand the impact of equalization better, as shown in Fig. 9 below.

When figures a and b are compared: to figures c and d, image improvement based on histogram equalization is counterproductive and thereby removed from the system. We moved on to the picture segmentation portion of the research after carefully analyzing the selected sample image from the hand motion collection. In the picture segmentation phase, we attempted two thresholding algorithms and a one-color space segmentation approach, which are explained further below. We attempted to segment a hand gesture image by binarizing it and repeatedly looking for the optimal threshold to segment the image and eliminate the background.

Figure 9 above shows the result obtainable from image segmentation using different thresholding functions. The yen and Otsu thresholds retain most of the information in the image. The section below tries both yen and Otsu thresholding methods for image segmentation. Figure 9a shows the outcome of the Otsu threshold, whereas Fig. 9b shows the yen threshold.

We tried different color segmentation approaches to remove the background from the foreground. The first approach is for image segmentation based on the upper and lower blue ranges in the HSV space, and the result is shown below. Our final approach in hand segmentation uses an iterative approach to select the best Hue, Saturation, and values in the sample image. This method proved the most effective and yielded the best result, as illustrated in the image below.

As we can see in the image above, the region of interest most needed for the hand gesture recognition task is highlighted above, and the background has been removed. We can now loop through our images and apply the image segmentation algorithm we created based on the HSV color space. In the next stage, we discussed how different Machine Learning results were obtained from the project.

### Hand Gesture Recognition

**Fig. 10:**
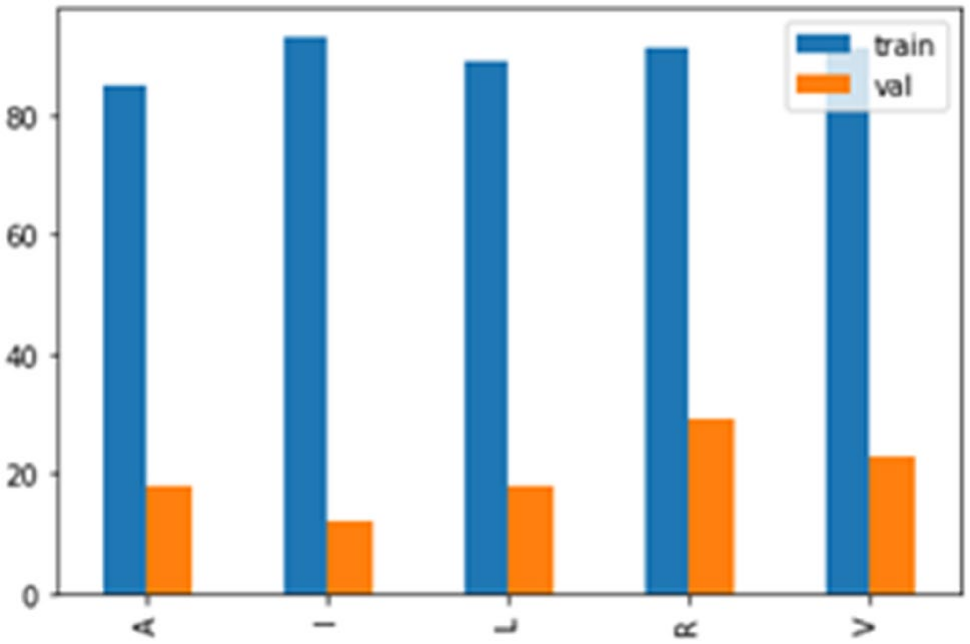
Train and Validation Class

The first step in the machine learning stage of the project is to load all image datasets into our system and carefully explore the dataset to understand the training and validation data distribution (Fig. 10).

For the hand gesture prediction, we tried classifying the unsegmented image first and then compared the results of the two models. We observed that the performance of our model almost doubled because of image segmentation. We achieved an accuracy of 45 percent using the unsegmented images and an accuracy of 58 percent using the unsegmented images. However, the system did not achieve the optimal result due to the quality of sample images and the number of images used. The images and table below give a summary of the models used in this project. The author has also identified areas for improvement, highlighted in this project’s conclusion section.

**Fig. 11:**
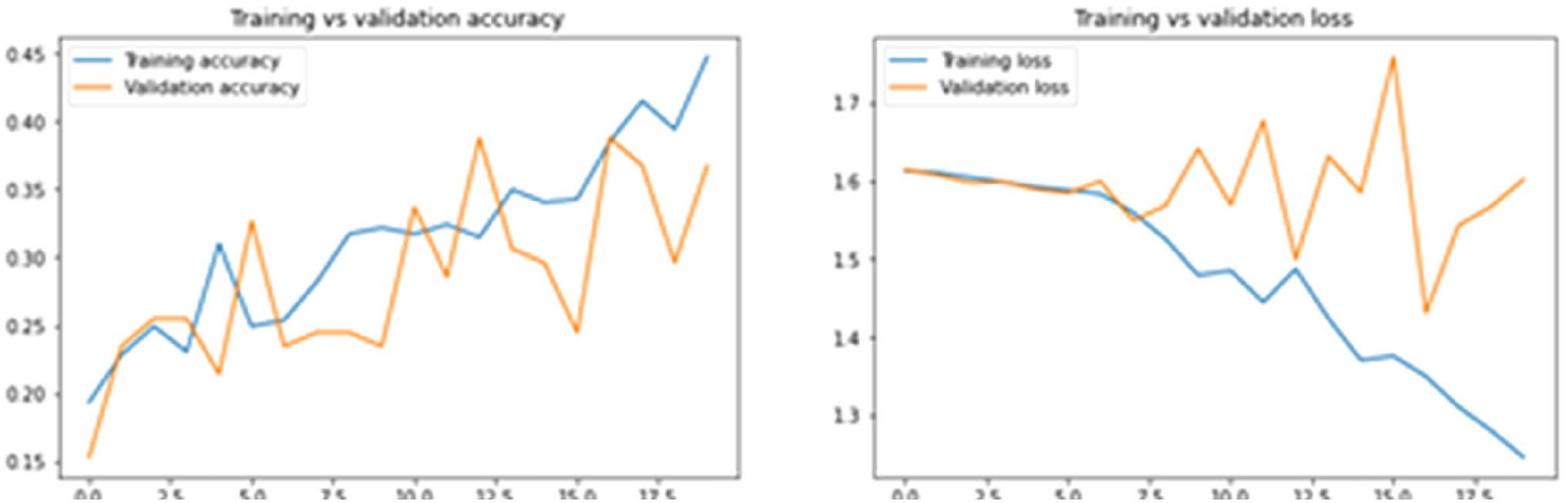
Accuracy and Loss before Image Segmentation

**Fig. 12:**
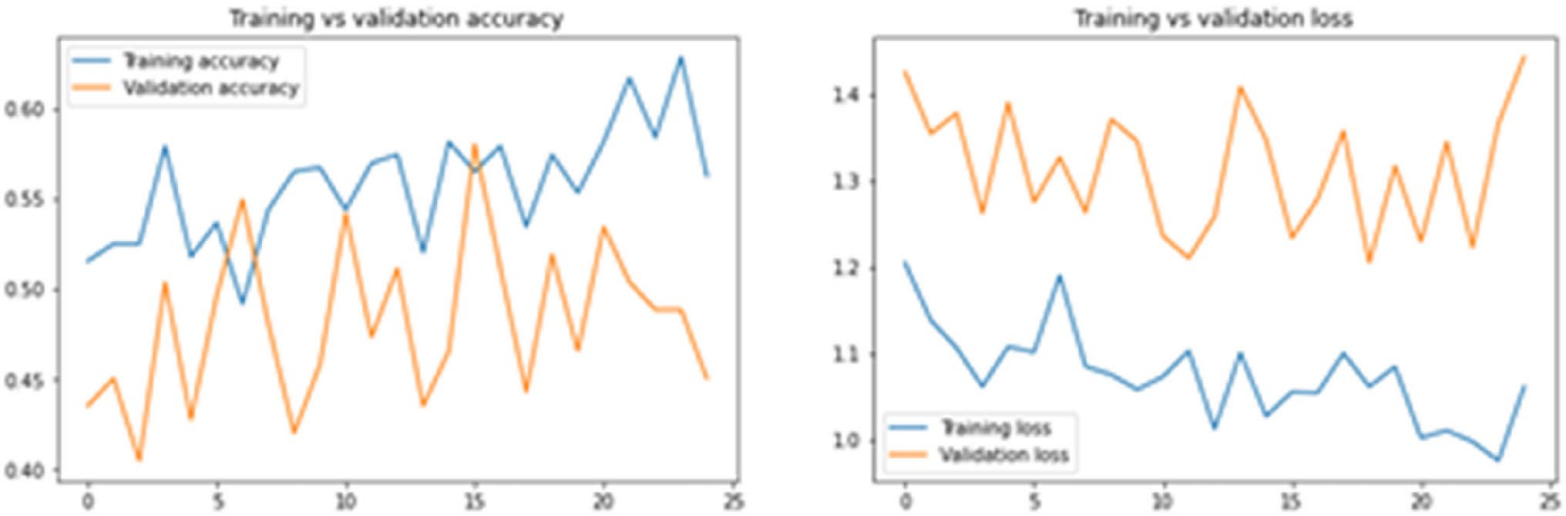
Accuracy and Loss after Image Segmentation

The accuracy and loss for both training and validation images before and after image segmentation is given in Figs. 11 and 12. Moreover, Table 1 illustrates the confusion matrix summary obtained by predicting the set of images under different lighting conditions.

**Table 1:** Performance Metrics for the two-model used

Sign	Precision	recall	f1 score
First model before segmentation			
A I	0.40	0.67	0.50
L R V	0.67	0.50	0.57
	0.50	0.25	0.33
	0.50	0.75	0.60
	0.62	0.70	0.62
Second model after segmentation			
A I	0.44	0.92	0.62
L R V	0.50	0.25	0.33
	0.25	0.25	0.25
	0.75	0.75	0.75
	0.84	0.25	0.40

## Discussion

This project extensively studied computer image processing and analyzed various literature and techniques. As a result, the author is familiar with the different approaches and algorithms required for image classification and hand gesture recognition. The summary of the discoveries by the author in this research project is illustrated below. While it is critical to analyze our data and pre-process photographs for better results, no approach exists that works on all images and image types. Therefore, the data scientist or image processor must be able to accurately analyze the picture to choose the optimum image enhancement for the image and the job. As we can see from the above result, the image-enhancing processes were unproductive and did not provide a satisfactory outcome.

### Recommendations

Various factors influence image quality. The camera type we used to take the photographs, the direction and angle of capture, the lighting condition, the skin tone, and other characteristics are all likely to have an impact on the image processing and hand motion identification job. Unlike a straightforward Machine Learning classification or regression task, which makes it easier to fix the features for the prediction of the target class, deep learning classification has several hyper-parameters and criteria for the picture to be usable. For example, the image's form and size must be as specified by the algorithm of choice. Nonetheless, biased and racial results from hand gesture detection systems may have risky and uncontrolled consequences. Furthermore, we have to compare analytical, discriminating tendencies to various advantages to provide a fair model.

### Ethical Considerations

If the true goal of a hand gesture recognition system is to deal with sign language efficiently, then all the multiple and diverse elements of sign language should be considered, which means that sign language must be researched in full and sign language recognition systems fully implemented. In this sense, scholars working on sign language must take an interdisciplinary approach with the assistance of the speech-impaired community and experts. Numerous studies gave scant attention to the effect of including or excluding specific words from the task of hand gesture recognition.

The implications of this omission may be detrimental to users who rely on such systems. Another oversight in hand gesture recognition systems is the omission or under-representation of some demographics or groups in the training dataset, which results in a biased system that overgeneralizes the training data. This could result in deploying machine learning systems that fail to perform efficiently, when used by an underrepresented user. Since the image's luminous level significantly affects the vision-based hand gesture recognition system, this can result in systems malfunctioning when employed in light conditions not included in the training data.

## Conclusion

The project is a rudimentary static gesture recognition system that cannot do dynamic gesture recognition tasks. Since the dataset utilized is not diverse (the author’s hand gesture photographs), the system may fail when applied to a different dataset. The picture obtained was taken using a simple camera and is of poor quality. The technology is sensitive to lighting conditions and may not perform optimally on a picture with varying lighting conditions. Through the study, the research team uncovered other methodologies that are worth examining. First, several picture-enhancing algorithms must be researched and applied in future work. Second, feature extraction, such as the silhouette image-based technique, should be studied in future research. Third, optimizing, selecting, and weighting processing of extracted characteristics will be investigated to simplify computations. In addition, the algorithm design element will examine the recognition accuracy and robustness, ease of use, and operation efficiency. Finally, future studies should incorporate more modern technology and approaches, such as tracking to allow dynamic gesture detection and learning the newest technology in the sector to enhance the metric’ performance.

The hand gesture recognition project in this article was produced using skin color segmentation in the HSV color space. This algorithm uses robust skin color segmentation properties in the HSV space to counteract the impact of changes in illumination conditions on gesture detection. In addition, several image-enhancing procedures were performed: on the image prior to hand segmentation. The hand gesture orientation was generalized after the Region of Interest was segmented using the data generator function for batch gradient descent. These processes mitigate the effect of variations in gesture orientation on gesture recognition. The generalization ability of the algorithm is improved during the gesture recognition stage by integrating the embedded deep sparse auto-encoders in the classifier. The experimental findings reveal that, following segmentation, the suggested technique is robust and considerably preferable to the other method in classification performance and recognition consistency.

## Figures and Tables

**Fig. 13:**
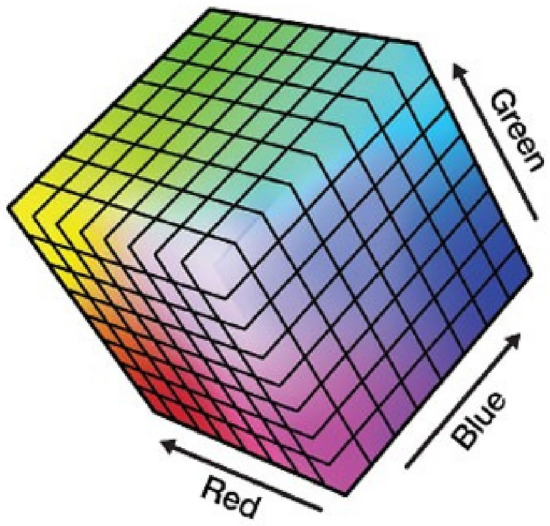
RGB color space

**Fig. 14:**
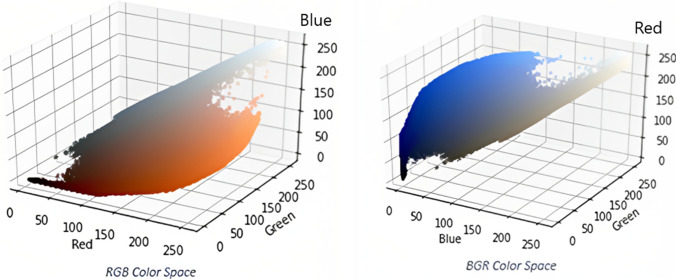
RGB and BGR color space

**Fig. 15:**
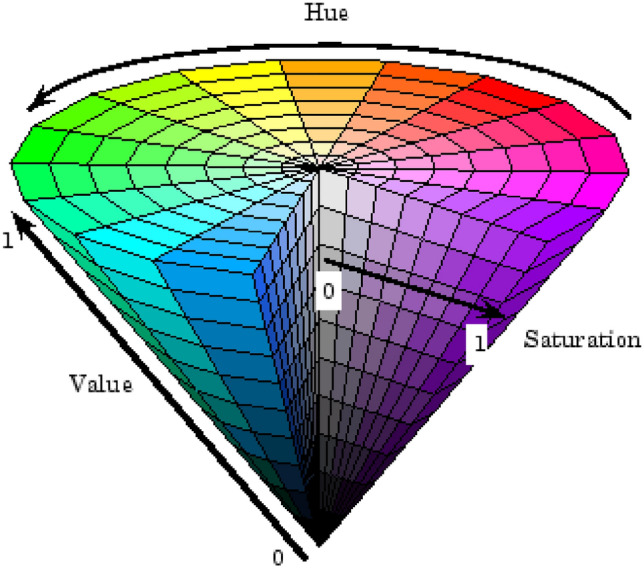
HSV color space

**Fig. 16:**
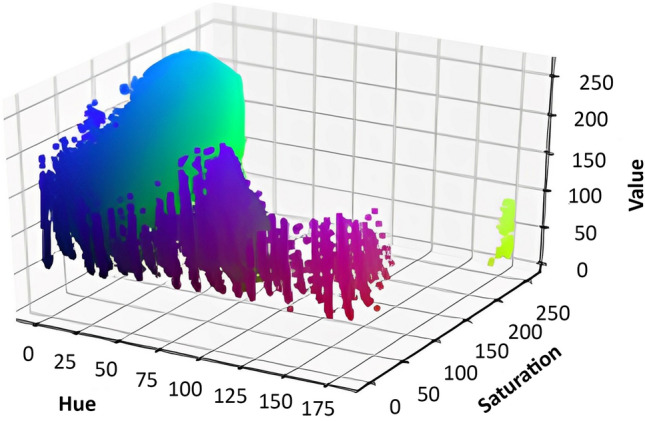
HSV color space of a sample image

**Fig. 17:**
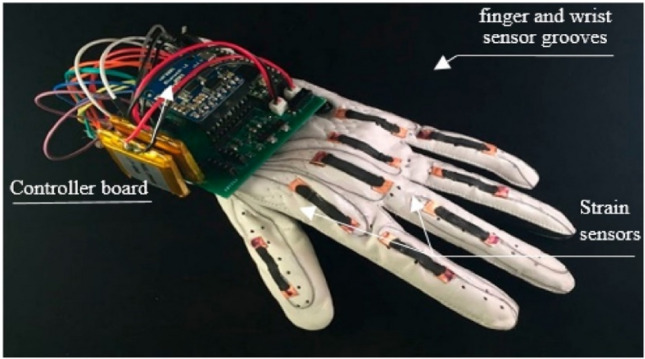
Wearable data glove

**Fig. 18:**
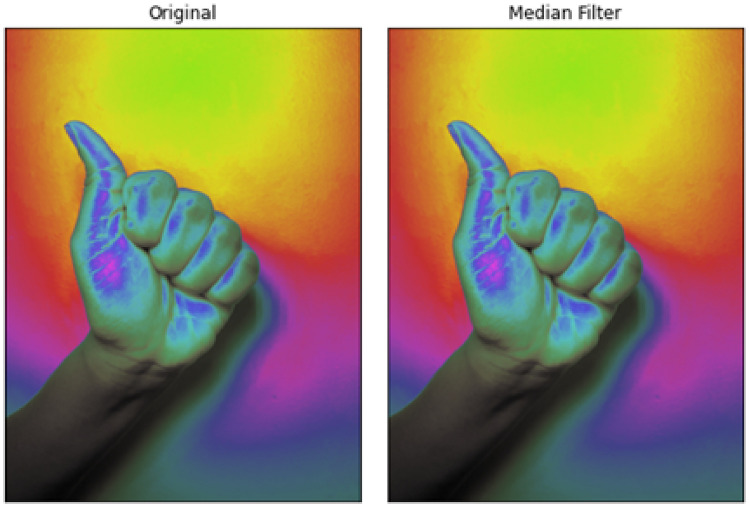
Median filter of a sample hand gesture

**Fig. 19:**
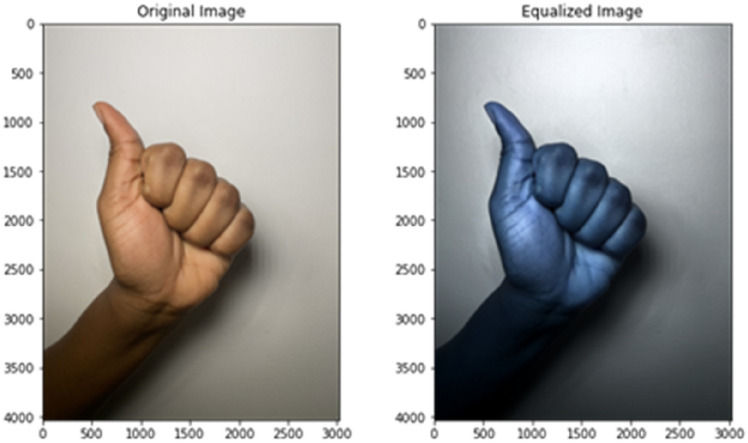
Effect of histogram equalization on a sample hand gesture

## Data Availability

The data used to support the findings of this study are available from the authors upon request. The data are not publicly available due to the presence of sensitive biological information that may compromise the privacy and confidentiality of research participants.

## Appendix 1: Color Space

Figs. 13, 14, 15 and 16.

## Appendix 2: Hand Representations

Figs. 17, 18 and 19.
